# New Insight for the Diagnosis of Gastrointestinal Acute Graft-versus-Host Disease

**DOI:** 10.1155/2014/701013

**Published:** 2014-03-11

**Authors:** Florent Malard, Mohamad Mohty

**Affiliations:** ^1^Service d'Hématologie Clinique, Centre Hospitalier et Universitaire (CHU), CHU Hôtel-Dieu, Place Alexis Ricordeau, 44093 Nantes, France; ^2^Université Pierre et Marie Curie, INSERM, UMRs 938, 184 rue du Faubourg Saint-Antoine, 75012 Paris, France; ^3^Service d'Hématologie Clinique et de Thérapie Cellulaire, Hôpital Saint Antoine, APHP, 75012 Paris, France

## Abstract

Allogeneic stem cell transplantation (allo-SCT) is a curative therapy for different life-threatening malignant and nonmalignant hematologic disorders. Graft-versus-host disease (GVHD) remains a major source of morbidity and mortality following allo-SCT, which limits the use of this treatment in a broader spectrum of patients. Early diagnostic of GVHD is essential to initiate treatment as soon as possible. Unfortunately, the diagnosis of GVHD may be difficult to establish, because of the nonspecific nature of the associated symptoms and of the numerous differential diagnosis. This is particularly true regarding gastrointestinal (GI) acute GVHD. In the recent years many progress has been made in medical imaging test and endoscopic techniques. The interest of these different techniques in the diagnosis of GI acute GVHD has been evaluated in several studies. With this background we review the contributions, limitations, and future prospect of these techniques in the diagnosis of GI acute GVHD.

## 1. Introduction

Allogeneic stem cell transplantation (allo-SCT) is a curative therapy for different life-threatening malignant and nonmalignant hematologic disorders. In hematologic malignancies, the therapeutic efficacy of allo-SCT is due to the graft-versus-tumor (GVT) effect. However, the beneficial effect of GVT is counterbalance by the immunological recognition and destruction of cells and tissues of the recipient by the immune effectors of the donor, termed graft-versus-host disease (GVHD). GVHD remains a major source of morbidity and mortality following allo-SCT, which limits the use of this treatment in a broader spectrum of patients. Little progress has been made for the prophylaxis and the treatment of GVHD [1]. It is therefore essential to improve the management of GVHD, in both forms, acute and chronic. This requires a better understanding of the pathophysiology of GVHD in order to identify new therapeutic target and develop new immunosuppressive drugs.

Furthermore, early diagnosis of GVHD, particularly of acute GVHD, is often difficult because of the nonspecific nature of the associated symptoms and of the numerous differential diagnoses. These issues may lead to delay of the initiation of corticosteroids, which may have dramatic consequences for patients. Consequently several teams develop laboratory test to predict the risk of developing GVHD or responsiveness to treatment. Indeed the development of biomarkers in the allo-SCT setting is crucial, because the early identification of patients at high risk for GVHD has important therapeutic consequences, including more stringent monitoring and intensified prophylaxis of GVHD. Beside biological biomarkers, it is essential to develop new tools to aid in early diagnosis of GVHD and particularly of gastrointestinal (GI) acute GVHD, whose diagnosis can be particularly difficult. In recent years many progress has been made in medical imaging test and endoscopic techniques, and the interest of these different techniques in the diagnosis of GI acute GVHD has been evaluated in several studies. The aim of this work is, after recalling the clinical aspect and the current practice for the diagnosis of GI acute GVHD, to review the available evidence on these medical imaging test and endoscopic features for the diagnosis of GI acute GVHD; data on biomarkers have been extensively reviewed by Paczesny et al. [2, 3] and are not discussed here.

## 2. Clinical Aspect of Acute GVHD

Historically, GVHD was clinically divided as acute and chronic, according to the time of onset. Acute GVHD was defined as arising within 100 days after allo-SCT, whereas chronic GVHD occurs after 100 days after allo-SCT (with or without preceding acute GVHD). However, this distinction was not always so clear-cut, in particular after reduced-intensity conditioning, an increasingly used regimen [4, 5]. This led the National Institute of Health (NIH) to a new classification, including 2 new entities, late onset acute GVHD and overlap syndrome [6]. Late onset acute GVHD is defined as GVHD occurring after day 100, with clinical features of acute GVHD and no feature of chronic GVHD; the overlap syndrome is defined as GVHD with features of both acute and chronic GVHD. Acute GVHD is a clinicopathological syndrome involving mostly three organs: the skin, the gastrointestinal tract, and the liver. Any one organ or combination of these organs may be affected. Severity of acute GVHD is assessed by the severity of involvement of these three target organs, according to the classification described by Glucksberg et al. [7] and modified in 1995 [8], to include upper intestinal symptoms within the definition of acute GVHD and drop the use of the clinical performance score. Including late onset acute GVHD, up to 80% of patients will experience grades II–IV acute GVHD after allo-SCT with a match related or unrelated donor [9].

Skin is the most frequent organ involved in acute GVHD. Patients presented a typical maculopapular rash, pruritic, palmoplantar impairment is frequent at diagnosis, and the rash can spread to the whole body sparing the scalp. Skin detachment may occur in the most severe cases. Liver involvement of acute GVHD is first suspected on the biological test, patients have an elevation of serum bilirubin, and then patients presented icterus when bilirubin reached 30–50 *μ*mol/L. The diagnosis is often difficult as there are many other causes of liver dysfunction after allo-SCT, such as viral infection, iron overload, venoocclusive disease, sepsis, or toxic drug effects. A definitive diagnosis could be made by the examination of a liver biopsy; however, this is rarely performed due to the highly invasive character of the procedure. Thus the diagnosis of liver acute GVHD is a diagnosis of exclusion. The most common symptom of GI involvement of acute GVHD is diarrhea [10]. Anorexia or vomiting, alone or associated with diarrhea, is also considered as GI acute GVHD symptom [11], according to the revised Glucksberg classification [8]. Diarrhea in acute GVHD is secretory and usually voluminous, often reaching more than 2 L per day. In the most severe cases, patients presented abdominal pain and gastrointestinal bleeding may occur, reflecting the mucosal ulceration. Unfortunately, these symptoms are nonspecific and encountered in numerous differential diagnoses frequently observed after allo-SCT such as infection with* Clostridium difficile* colitis, viral infection (mainly cytomegalovirus (CMV)), drug toxicity, or neutropenic enterocolitis [12]. In the setting of umbilical cord blood allo-SCT these symptoms may also correspond to the cord colitis syndrome [13], related to an infection by a newly described bacteria,* Bradyrhizobium enterica *[14]. The diagnosis of GI acute GVHD is based upon the analysis of clinical and laboratory criteria in the appropriate clinical context after excluding other causes [15]. Thus bacteriological, virological, and parasitological stool culture, a search for* Clostridium difficile* toxin in stool, and virus DNA screening in plasma (cytomegalovirus (CMV), adenovirus (ADV), etc.) are usually performed in order to exclude other differential diagnoses. The gold standard in the diagnosis of GI acute GVHD is upper and lower GI endoscopy with histological validation [16]. However, this strategy has significant limitations. Indeed GI acute GVHD is characterized by a patchy distribution of the lesions, which can either affect a short segment of the small bowel or the entire digestive tract [17–21]. Given that esophagogastroduodenoscopy (EGD) and colonoscopy only explore a small segment of the small bowel, their diagnostic yield is limited. Also one should bear in mind that endoscopic abnormalities are usually found in a minority of cases (16–32%) [22] and are usually nonspecific. Consequently, additional biopsies are necessary, despite the lack of specificity of apoptotic bodies, the main histological hallmark, especially in the early phase after allo-SCT [23]. Finally endoscopic examinations and biopsies are relatively invasive in such fragile patients, often at high risk of bleeding in case of thrombocytopenia. In the last decade, several imaging modalities have been developed to offer extensive and noninvasive examination of the entire small bowel. The contributions of these techniques for GI acute GVHD diagnosis are reviewed here.

## 3. Medical Imaging Test

### 3.1. CT Scan and MRI

From the beginning of allo-SCT development, attempts have been made to use medical imaging to help diagnose GI acute GVHD. In 1988, plain abdominal radiography has been evaluated; 95% of patients with GI acute GVHD presented abnormal radiography with separation of bowel loop indicative of wall thickening, air fluid level, and small bowel dilatation; however, these features are not specific [24]. Consequently several studies have evaluated the contribution of computed tomography (CT scan) for the diagnosis of GI acute GVHD; the features most often found with CT scan are bowel wall thickening, abnormal mucosal enhancement, and bowel dilatation [21, 25–27]. Unfortunately, those signs are nonspecific. Indeed, similar radiologic finding is seen after allo-SCT in many other complications, such as infection with* Clostridium difficile* colitis, viral infection (mainly cytomegalovirus (CMV)), or neutropenic enterocolitis [21, 25]. However, some features may help differentiate GI acute GVHD from other GI complications of allo-SCT. Regarding bowel wall thickening, in GI acute GVHD, the thickening is generally moderate, whereas more severe thickening rather suggests* Clostridium difficile* or CMV colitis and neutropenic enterocolitis [25]. Furthermore, in GI acute GVHD, bowel wall thickening involved both small and large intestine in almost all cases; we can thus exclude* Clostridium difficile* colitis which affects only the large intestine [25]. Neutropenic enterocolitis also involves both small and large intestine; however, right colonic or caecum involvement present in 75–100% of neutropenic enterocolitis [25, 28] is uncommon in GI acute GVHD [25–27] and a discontinuous distribution of bowel involvement is more frequent in GI acute GVHD. Abnormal mucosal enhancement after administration of intravenous contrast material has been reported in up to 89% of patients with GI acute GVHD [25–27] and seems to be more common than in other GI complications after allo-SCT [25]. Some author reported the use of negative oral contrast material, leading to a lower rate of visualization of mucosal enhancement [27]. Furthermore, GI symptoms of GI acute GVHD often prevent the use of oral contrast material [11]. Regarding other radiological findings seen in GI acute GVHD; the incidence of bowel dilatation varies in an important way according to the studies, from 23 to 86% [25–27]. However, this remains more frequent than in other GI disorders after allo-SCT or in neutropenic patients [25]. Mesenteric infiltration, ascites, or blood vessel abnormality (engorgement of vasa recta) is also frequently observed after GI acute GVHD [25–27] and could help for the diagnosis.

Recently, Brodoefel et al. evaluate CT scan in early and late onset acute GVHD [26]. Results confirm that CT scan morphology of GI acute GVHD is independent of the delay between GI acute GVHD onset and the time of execution of the CT scan. The interest of this study lies on the development of a severity CT scan score based on 6 criteria to grade GI acute GVHD. This score correlates well with gut, overall clinical, and pathology grading. Thus CT scan lacks specificity for the diagnosis of GI acute GVHD; however, with an experienced user, it can provide valuable assistance for the diagnosis of GI acute GVHD. Use of oral contrast material is not recommended. Beside, one must pay attention to the nephrotoxicity of intravenous iodine contrast material, since renal function impairment is frequent after allo-SCT [29]. Furthermore, a CT scan score that correlates with clinical grading could be performed and provide valuable information regarding the evaluation of GI acute GVHD severity [26].

The use of magnetic resonance imaging (MRI) was reported only in a few cases [30, 31]. MRI findings are comparable to the CT scan features; in particular a bowel wall thickening associated with abnormal mucosal enhancement with gadolinium is reported. As for CT scan these features are not specific and do not permit discriminating GI acute GVHD from other etiologies. Thus MRI should not be performed for the diagnosis of GI acute GVHD; however, when MRI is performed after allo-SCT for another indication, these features should make us consider the diagnosis of GI acute GVHD.

### 3.2. US and CEUS

Findings with ultrasonography (US) are comparable to those of CT scan. Author reported bowel wall thickening and bowel dilatation [31–34]. Blood vessel abnormalities are also described using color Doppler imaging [33, 34]. Recently a prospective study evaluated US for GI acute GVHD diagnosis [35]. This study included 52 patients with GI symptoms after allo-SCT, 15 patients were lost to followup, 17 patients develop GI acute GVHD, and in 20 patients no GI acute GVHD was diagnosed (4 with chemo/radiotoxicity, one with* Escherichia coli* sepsis, and 15 without a specific diagnosis). US detects bowel wall thickening or bowel dilatation in 16/17 patients with GI acute GVHD. The sensitivity and specificity were, respectively, 94 and 95% in this study. However, one may question the control group, for GI acute GVHD diagnosis, and the interest of complementary investigation is to discriminate with infectious complication and neutropenic enterocolitis, whereas only 1 patient presented infectious GI complication in the control group. Thus, despite this study, US lacks specificity for GI acute GVHD diagnosis and we do not recommend its use in this setting.

On the other hand, data regarding new US techniques are much more promising. Recent progress has been made, with the development of contrast-enhanced ultrasound (CEUS). It is a real-time microvascular imaging technique, which use has been possible thanks to the development of novel echocontrast-enhancing agent [36]. These echocontrast-enhancing agents are gas-filled microbubbles, administered intravenously to the systemic circulation. CEUS has been used in active Crohn's disease [37], in which neovascularization of the small bowel wall has been described. Considering the role of neovascularization in the early stages of GI acute GVHD recently described [38], several teams evaluated the use of CEUS for the diagnosis of GI acute GVHD. After standard US, an ultrasound contrast agent is administered I.V. and CEUS is performed on the intestine. In 2011, Schreyer at al. reported that in contrast to Crohn's disease patients and healthy volunteers, patients with GI acute GVHD showed transmural penetration of microbubbles into the bowel lumen [39]. These results have been confirmed in 2 studies. The first study included 20 patients presenting GI symptoms after allo-SCT [40]. Out of 17 patients with biopsy proven GI acute GVHD, 14 showed penetration of the IV applied microbubbles into the bowel lumen, whereas in patients with viral or bacterial infection of the GI tract, no transmural penetration of the microbubbles was observed. The sensitivity, specificity, NPV, and PPV were, respectively, 82, 100, 81, and 100% in this study. The second study [41] compares CEUS finding in 14 patients with lower GI acute GVHD, 16 patients with only upper GI acute GVHD, and 4 patients with neutropenic enterocolitis. Transmural penetration of microbubbles into the bowel lumen was observed in all patients with lower GI acute GVHD and in one patient with neutropenic enterocolitis and was not reported in patients with only upper GI acute GVHD. Furthermore, in patients with lower GI acute GVHD, CEUS normalized after response to treatment, whereas, in corticosteroid resistant patients, it remained unchanged.

Overall, CEUS appears to be a sensitive method for assessment of GI acute GVHD, with a very high specificity from 75% to 100%; however, there were very few patients in the control group, 3 in one [40], and in the other 4 (only patients with neutropenic enterocolitis were clinically relevant) [41]. Finally, CEUS is a noninvasive promising tool for assessment and monitoring of GI acute GVHD, restricted to patients with lower GI acute GVHD. Furthermore, it requires specific device and a highly trained physician able to perform the procedure, not available in many hospitals. Prospective studies are needed to confirm those results.

### 3.3. PET/CT

**Figure 1:**
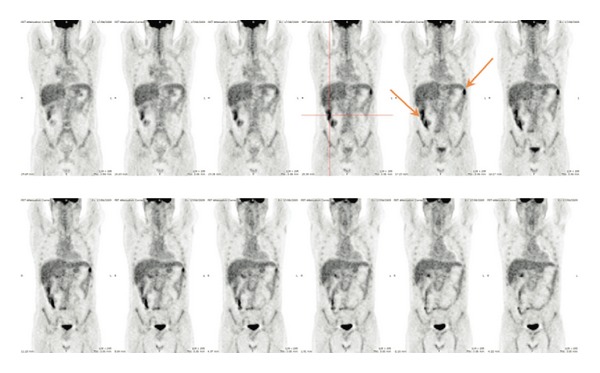
Example of a true positive patient: ^18^F-FDG PET/CT performed 25 days after allo-SCT in a 46-year-old patient who presented with signs of acute GI-GvHD 30 days after allo-SCT.

^18^F-Fluorodeoxyglucose positron emission tomography (^18^FDG-PET/CT) is widely used in haematological malignancies and solid tumours [42, 43]. More recently ^18^FDG-PET/CT has been found to be useful in detecting inflamed areas throughout the entire intestinal tract in inflammatory bowel diseases [44]. Furthermore, two preliminary case reports suggest that ^18^FDG-PET/CT is appropriate to assess the exact localization of GI acute GVHD and to evaluate treatment response [45, 46]. These observations have led to the evaluation of ^18^FDG-PET/CT for the diagnosis of GI acute GVHD in prospective studies. Stelljes et al. [47] demonstrated in 2008 that GI acute GVHD was visualized by ^18^FDG-PET/CT in a murine model and translated these results in a cohort of 30 patients with diarrhoea and suspected lower GI acute GVHD. The diagnosis of GI acute GVHD was based on histologic finding. No increased ^18^FDG was detected in 13 patients without histologic evidence of GI acute GVHD, whereas 14 of 17 patients with biopsies proven GI acute GVHD showed significant ^18^FDG uptake in the gut. The Se, Sp, NPV, and PPV were, respectively, 82, 100, 81, and 100%. A secondary ^18^FDG-PET/CT was performed in some patients and a significant decrease of ^18^FDG uptake was observed in patients' responder to GI acute GVHD therapy. Recently we published the results of a prospective study evaluating the predictive value of ^18^FDG-PET/CT for early diagnosis of GI acute GVHD in 42 patients [48]. ^18^FDG-PET/CT was systematically performed at a median of 28 days (range: 24–38) after allo-SCT and GI acute GVHD onset was monitored within 4 weeks after completion of ^18^FDG-PET/CT. Among the 10 patients who presented GI acute GVHD, 9 had a positive ^18^FDG-PET/CT (Figure 1). Regarding the 32 patients without GI acute GVHD, only 5 patients had a positive ^18^FDG-PET/CT. The Se, Sp, NPV, and PPV were 81, 90, 96, and 60%, respectively.

^18^FDG-PET/CT appears to be a noninvasive, sensitive, and very specific biomarker for GI acute GVHD diagnosis in patients with diarrhoea [47] and may help to monitor the response to corticosteroids [45, 47]. Furthermore ^18^FDG-PET/CT can detect inflammatory activity of the GI tract associated with subclinical GI acute GVHD before clinical symptoms onset [46, 48] in contrast to CT scan, MRI scan, or US, where radiological features occur later. Finally, data remains limited so far and larger prospective studies seem indispensable before using ^18^FDG-PET/CT in routine in this setting. The development of new PET tracers targeting apoptosis, one of the histological hallmarks of GI-aGVHD [49], is very promising.

## 4. Endoscopic Examination

### 4.1. Wireless Video-Capsule Endoscopy (WCE)

**Figure 2:**
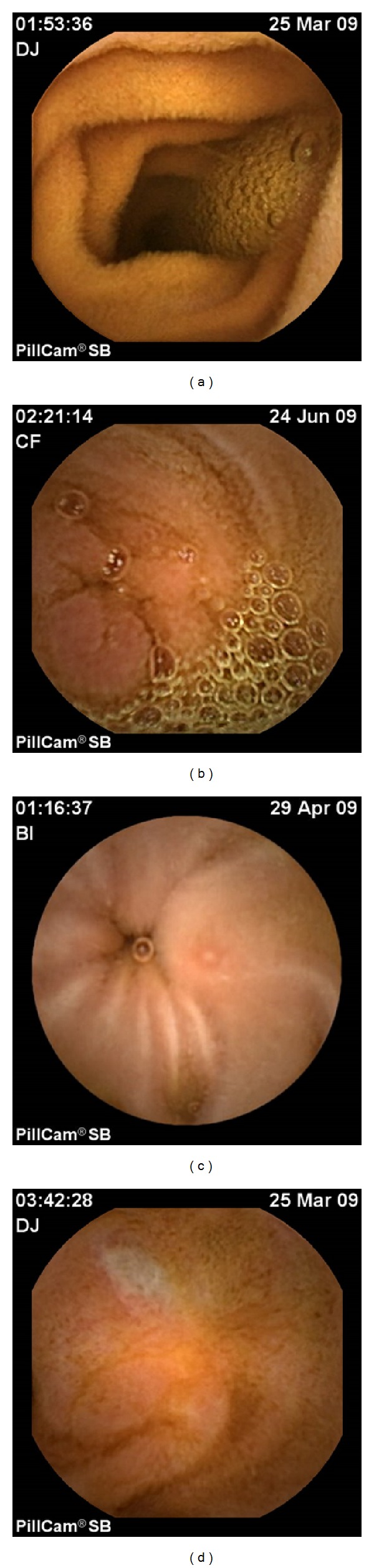
Small bowel lesions of GI-GVHD detected by wireless capsule endoscopy (WCE). (a) Normal jejunum, (b) focal edema and enanthematous aspect of the jejunum, (c) aphtoid erosion in the proximal ileum, and (d) large superficial ulceration of the ileum.

Wireless video-capsule endoscopy (WCE) is a sensitive, noninvasive diagnostic tool for the exploration of the small intestine. WCE is routinely used for the diagnosis of anemia and occult bleeding and Crohn's disease and recognition of intestinal tumors [50]. Given that GI acute GVHD is characterized by a patchy distribution of lesions, which can either affect a short segment or involve the whole gastrointestinal tract, WCE that explores the whole small intestine is a seductive approach for GI acute GVHD diagnosis. Several studies evaluated WCE in adults [20, 51–54] and children [55, 56] with GI acute GVHD symptoms. These studies highlight the heterogeneity of the involvement of the small bowel; in some patients WCE shows lesion of the whole small bowel, whereas in others lesions are discontinuous, sparing some area of the small bowel. Regarding lesions observed, author reported edema, erythema, erosion, ulceration, and bleeding (Figure 2). A delayed gastric transit time was also reported [52, 54]. It should be noted that intestinal stenosis contraindicated WCE. Fortunately intestinal stenosis is exceptional and occurs in patients with a long history of refractory GI acute GVHD. We highlight that no retention of the capsule in the small bowel was reported in the setting of GI acute GVHD. Yakoub-Agha et al. evaluated WCE in 10 patients with suspected GI acute GVHD and results were compared with EGD and duodenal biopsies [51]. Five patients had a normal WCE examination; EGD and duodenal biopsies were also normal in those five patients. Four patients were successfully treated symptomatically and one patient died from toxoplasmosis. Regarding the five remaining patients, WCE disclosed GI acute GVHD lesion, whereas EGD was considered as normal in 2 of them and duodenal biopsy in one of them. GI symptoms improved in all of these patients after adjustments of their immunosuppressive treatment. The contribution of WCE for the adaptation of immunosuppressive therapy in patients with suspected GI acute GVHD and the apparently high NPV in this study is very appealing. Neumann et al. also investigated the role of WCE in 14 patients with clinical symptoms of GI acute GVHD [20]. Only 13 patients could be evaluated, given that the capsule remained in the stomach and was removed endoscopically in one patient. In all 7 of 13 patients with histologically confirmed GI acute GVHD, WCE reveals typical signs of GI acute GVHD, whereas EGD reveals sign of GVHD in only 4 patients. In all 6 remaining patients, EGD, histology, and WCE were normal but in one patient WCE showed erosive enteritis. Here to, the NPV of WCE is very high, with better sensitivity than EGD. However, biopsies cannot be performed during WCE, and despite the apparently high NPV in these studies, they are based on a very low number of patients and EGD with biopsies remains indispensable. Furthermore, WCE probably lacks specificity given that erosion and ulceration were reported on WCE performed in patients with CMV diseases after allo-SCT [52, 57]. Overall, WCE realization can be useful in patients with GI acute GVHD symptoms and normal EGD. Furthermore, a visual grading of GI acute GVHD lesion has been performed in several studies [20, 51] according to the Brand criteria [58] as follows: grade 0, normal; grade I, focal erythema; grade II, moderate or diffuse erythema, nodularity; grade III, erosion and or vulnerable mucosa; and grade IV, ulceration, denuded mucosa, and bleeding. It could be useful to evaluate the severity of GI acute GVHD and manage immunosuppressive treatment. Finally, one limit of WCE for the investigation of GI acute GVHD is the bowel preparation required [59]. Indeed, despite it being noninvasive, patients had a 12-hour overnight fast and drank 1 liter of a polyethylene glycol-electrolyte (PEG) solution 2 hours prior to swallowing the WCE. The absorption of the PEG may be difficult in frail patients with upper gastrointestinal symptoms and limits WCE use in this setting.

### 4.2. Endoscope-Based and Probe-Based Confocal Laser Endomicroscopy (eCLE and pCLE)

Regarding new procedure in endoscopic examination, another interesting method is confocal laser endomicroscopy, a high-resolution imaging modality, allowing access to* in vivo *histology at the subcellular level during ongoing endoscopy. Confocal laser endomicroscopy, either using an endoscope-based (eCLE) or a probe-based technology (pCLE), aims to decrease the number of standard biopsies and their associated risk, by providing real time* in situ* microscopy [60]. CLE requires the IV injection of a fluorescent contrast agent, fluorescein, allowing vasculature and cellular architecture to be better appreciated. In addition acriflavine could be administered topically providing staining of cell nuclei, not stained by fluorescein. Established CLE applications include the diagnosis of Barrett's oesophagus, gastric intestinal metaplasia coeliac disease, and microscopic colitis [61]. There is very few data regarding the use of CLE in GI acute GVHD. Apoptotic bodies on histological lesion is one of the hallmarks of GI acute GVHD and the eCLE equivalent of an apoptotic cell is 100% nuclear staining with topical acriflavine. Thus Bojarski et al. examined endomicroscopy features and conventional histology on targeted biopsies of sigmoid and rectum in 35 patients with acute diarrhea after allo-SCT [62]. In 16 patients, eCLE and histology showed no evidence of GI acute GVHD. In 19 cases, there was evidence of GI acute GVHD on conventional histology. In 14 of these 19 cases, the diagnosis of GI acute GVHD could be already performed by eCLE during the procedure. Endomicroscopy showed single cell apoptosis within the crypt epithelium in histologically proven grade 1 GI acute GVHD. Other features seen at endomicroscopy for grades 2–4 include apoptosis of entire crypts, focal destruction of crypt, and capillary leakage of fluorescein; in severe case (grade 4 GI acute GVHD), near complete destruction of the colonic crypt with flat mucosa was observed. In the 30 controls (15 infectious colitis and 15 ulcerative colitis), eCLE showed inflammatory changes but no evidence of GI acute GVHD. Altogether, Se of eCLE was 74% and Sp was 100%. Although this study is limited to lower GI acute GVHD, data suggest that CLE can be used to diagnose upper GI acute GVHD. Thus Hundorfean et al. reported the diagnostic of gastric acute GVHD utilization of fluorescein guided CLE [63].

**Figure 3:**
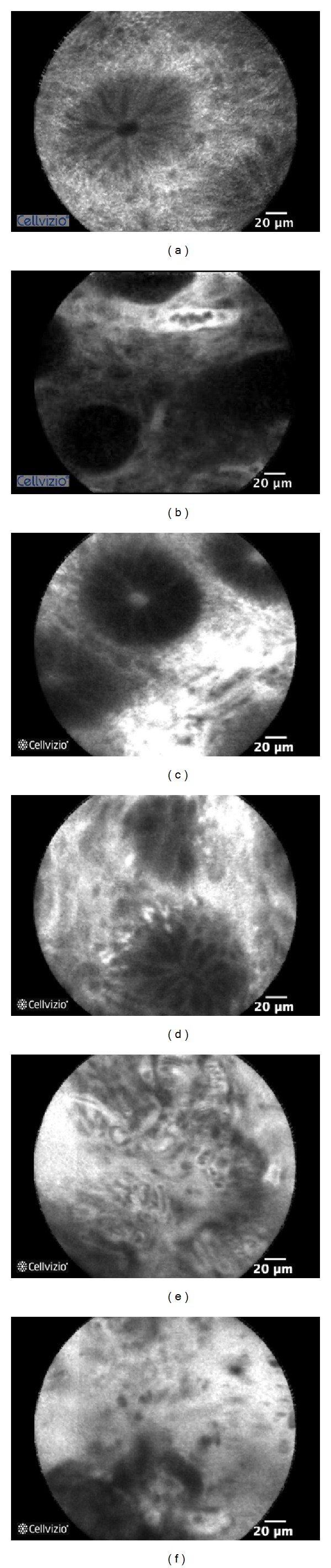
Probe-based confocal laser endomicroscopy (pCLE) patterns observed in GI-GVHD patients following intravenous injection of fluorescein. (a) Normal sigmoid, (b) abnormal microvessel network with dilation of a microvessel surrounding a colonic crypt (mild GVHD), (c) mild increase in fluorescein intensity in the lamina propria (mild GVHD), (d) distorted crypts (moderate GVHD), and ((e), (f)) major architectural changes showing proliferation and dilation of microvessels in the lamina propria, major fluorescein extravasation, and complete destruction of the colonic crypt architecture (severe GVHD).

More recently our team evaluated WCE and pCLE to predict the risk of GI acute GVHD in early stage asymptomatic patients [64]. 15 patients were prospectively examined with a WCE and duodenal and colorectal pCLE and underwent standard biopsies between day 21 and day 28 following allo-SCT independently of the presence or absence of digestive symptoms. In the current study pCLE was used after IV injection of fluoresceine and we were not able to study the nuclei. However, our study focused on the vascular compartment and dynamic changes, since the role of neovascularization in the early stages of GI acute GVHD has been recently highlighted [38]. Furthermore, acriflavine is not approved for use in France and in many other countries. During follow-up period, 8 patients developed acute GVHD. pCLE accurately identified 7 out of 8 patients who developed acute GVHD during followup (Figure 3). pCLE presents mild anomaly in 2 patients who did not develop any sign of acute GVHD. The Se, Sp, NPV, and PPV of pCLE to predict the onset of acute GVHD were 87, 71, 77, and 83%, respectively.

Overall, CLE allows rapid diagnostic of GI acute GVHD with high accuracy while performing endoscopy. Bojarski et al. reported a Sp of 100% and discriminated very accurately GI acute GVHD from infectious complications [62]. Once GI acute GVHD has been diagnosed* in vivo*, unnecessary biopsy could be avoided, in these frequently thrombocytopenic and at high risk of bleeding patients. Furthermore, pCLE allows very early detection of lesions suggestive of GI acute GVHD before the onset of symptoms, which probably reflect the global alloreactivity in the body. Of course, larger prospective studies seem indispensable to confirm these promising results. This confirmation is indispensable before being able to recommend the use of this technique in routine. Furthermore, it requires specific device and experienced users, which are not yet available in all centers.

## 5. Conclusion and Perspective

Achievements have been made regarding the development of new tools to assess the diagnosis of GI acute GVHD. Advantages, limitations, future developments, and recommendations regarding the use of each technique are summarized in Table 1. The contribution of conventional imaging techniques, CT scan, MRI scan, and US, is limited. Their use is not recommended in clinical practice even if in some situations they could be valuable. Data regarding the new imaging technique, ^18^FDG-PET/CT and more particularly CEUS, are more promising, even if further prospective studies are warranted to validate their use in clinical practice. However, starting today, their use may be considered in some patients where the diagnosis of GI acute GVHD could not be achieved and to evaluate the response to corticosteroids. Regarding WCE its use should be considered in patients with GI acute GVHD symptoms and normal upper and lower endoscopy—or in case of contraindication—in order to explore the whole small bowel. Finally,* in situ* histology using CLE is probably the future, to avoid biopsies in thrombopenic patients and make an immediate diagnosis, enabling the clinician to begin corticosteroids without delay.

## Figures and Tables

**Table 1:** Advantages, limitations, future developments and recommendations for the use of each technique.

Techniques	Advantages	Limitations	Future developments	Recommendations
CT scan	Easily available Noninvasive	Lack of specificity Irradiant Nephrotoxic	Not planned	Eventually

MRI	Nonirradiant Noninvasive	Lack of specificity Expensive Hardly available	Not planned	Not recommended

US	Noninvasive Easily available Cheap	Lack of specificity	Not planned	Not recommended

CEUS	Sensitive Specific Noninvasive	Limited to lower GI acute GVHD Specific device Trained physician	Prospective studies	Not available in routine

PET/CT	Sensitive Specific Early detection Noninvasive	Expensive Irradiant	Prospective studies	Useful in some patients

WCE	Noninvasive Sensitive	Bowel preparation Lack of specificity	Prospective studies	Useful in some patients

eCLE and pCLE	Sensitive Specific Early detection	Invasive Specific device Trained physician	Prospective studies	Not available in routine

CT scan: computed tomography scan; MRI: magnetic resonance imaging; US: ultrasonography; CEUS: contrast-enhanced ultrasound; GI acute GVHD: gastrointestinal acute graft-versus-host disease; WCE: wireless video-capsule endoscopy; eCLE: endoscope-based confocal laser endomicroscopy; pCLE: probe-based confocal laser endomicroscopy.
